# Empowering youth as Changemakers for gender-transformative action: a play-based participatory approach in South-East Nepal

**DOI:** 10.1177/17579139241287673

**Published:** 2024-10-14

**Authors:** S Begg, B Jha, N Shah, A Shrestha, J Pulford, S Parker

**Affiliations:** Liverpool School of Tropical Medicine, Liverpool L3 5QA, UK; Tribhuvan University, Kathmandu, Nepal; Sabal Nepal, Rajbiraj, Nepal; Nepal Cricket Foundation, Biratnagar, Nepal; Liverpool School of Tropical Medicine, Liverpool, UK; Liverpool John Moores University, Liverpool, UK

**Keywords:** youth participatory action research, gender norms, health inequalities, capacity building, empowerment, adolescent health

## Abstract

**Aims:**

This study explored how youth participatory action research (YPAR) methods, specifically play-based activities integrated with cricket, can engage adolescent girls in Nepal’s Terai region to identify and address gender-related health challenges. It aimed to assess how these methods contribute to empowering girls, developing research capacities, and promoting gender equity through sports.

**Methods:**

Three interactive workshops were held with adolescent girls forming ‘Cricket Changemakers’ teams. Through a combination of cricket-based activities, participatory games, and discussions, the workshops aimed to build research skills and explore gender issues in their communities. Qualitative data were gathered through observations, reflective notes, and outputs from play-based tasks. Data were analysed using framework analysis to evaluate the contribution of these activities to key YPAR processes such as power sharing, communication, and strategic thinking.

**Findings:**

The play-based methods improved collaboration, networking, and communication among participants. The cricket activities fostered collective power and provided opportunities for girls to reflect on and challenge local gender norms. Participants identified issues including restrictions on mobility, societal expectations, and the unequal distribution of household chores. The workshops facilitated the development of research action plans focused on increasing girls’ participation in cricket and shifting community perceptions of girls’ roles.

**Conclusions:**

The integration of play-based YPAR methods shows promise for fostering gender-transformative change among adolescent girls in Nepal. These methods created a space for girls to express challenges and co-develop strategies for social change. Findings suggest that sports-based participatory research can be a valuable tool in public health interventions for gender equity, though more work is needed to address methodological challenges and ensure meaningful participation.

## Introduction

Gender is a powerful determinant of health outcomes in Nepal, as it is across the world. As a determinant of health, gender operates across multiple layers of influence, from the individual to the societal level. Personal beliefs, interpersonal relationships, community structures, and societal norms all significantly influence each other and shape access to resources, define acceptable behaviours, and differentiate exposure to risks, impacting physical, mental, and social wellbeing.

Nepal’s cultural context is deeply influenced by patriarchal values, where traditional gender roles assign men as primary breadwinners and decision-makers, while women are expected to manage domestic responsibilities. As a result women’s autonomy in healthcare decision-making is often limited by male dominance, leading to suboptimal health outcomes.^1,2^ Similarly, rising rates of reports of experiences of intimate personal violence by women in Nepal (from 14% to 17% between 2016 and 2022) reflects and reinforces both interpersonal and societal norms that shape gender roles and power dynamics,^3–5^ while expectation to conform to gender-based behaviours and isolation postmarriage means women and adolescent girls are more depressed, and more anxious, than their male peers.^6–8^

Working with adolescents in gender transformative approaches to health promotion is critical because this period marks a significant life phase in identity formation and attitude development towards gender roles.^9–11^ Engaging adolescents through sports offers a unique opportunity to address and reshape harmful gender norms when individuals are most open to change. Past research supports the effectiveness of sports-based interventions, such as community cricket programmes in India which successfully reduced acceptability of gender-based violence among young adolescent boys.^12,13^ However, critical examination of extracurricular sports programmes highlights that interventions must not only promote gender equity but also engage with the ‘hidden curriculum’ in sports education, which may in fact reinforce harmful gender stereotypes.^14,15^ The use of sports must, therefore, be purposeful.

Youth participatory action research (YPAR) is a transformative approach to research, which aims to engage young people as important agents in the research process. With a focus on principles of equity and experiential knowledge, YPAR is action orientated. The collaborative research-to-action approach means that it can be a powerful tool in developing relevant, locally grounded solutions to public health problems, including those at the intersection of gender and health.^16–18^ YPAR recognises the power dynamics within communities and aims to strengthen and mobilise protective social norms while envisioning new ways of working. Finally, with the focus on action and effecting real-world change,^19^ YPAR not only aims to inform policy and practice but also develops the critical consciousness of youth, empowering them to become agents of change.^20^

Projects applying YPAR have shown promising outcomes related to gender and health, engaging adolescents in addressing health issues through participatory research processes.^21–23^ While their application in the intersection of sports, gender, and health are limited, there are indicators that this approach is adaptable for interventions within sports and development contexts.^24,25^ Such participatory research is increasingly recognised as a key tool to empower youth, with growing calls to effectively involve youth in critical engagement in sports and development programmes.^26^

There are, however, major challenges in actioning YPAR in sport and development contexts. While there is a growing emphasis on ‘youth voice’ in the form of youth advisory groups or boards,^27–29^ much of this is unevaluated or under reported, omitting details on methods used or outcomes and impact. Furthermore, key tensions have arisen around the disconnect between the activity preferences of youth engaged as research participants – playing sport – and research activities used by co-researchers such as tasks that require sitting down and writing.^30^

A way forward for addressing this method-context disconnect is to look to Youth-Creative Action Research (Y-CAR), which has incorporated spoken word, hip hop, and digital media creation, centring on forms of expression embraced by youth to explore and address social challenges.^31^ Furthermore, play- and game-based methods in YPAR are often limited to research with younger children; however, when they have been used, play has been found to create a fluid and playful research environment that blurred the lines between play and work,^32^ and by speaking the ‘language of childhood’ these methods can give children a voice in research.^33,34^

Ultimately, while there is clearly an important opportunity to mobilise the strengths of YPAR in the context of sport and development, including to achieve positive changes in health outcomes and the social determinants of health, progress must be made on how to operationalise this. First, to avoid key pitfalls around YPAR such as concerns around tokenism, which the under scrutinised ‘youth voice’ structures in sports and development may attract if their outcomes and impact continue to go unreported. This includes enhancing key processes for YPAR: training and practice of research skills, promoting strategic thinking, group work, opportunities for networking, communication, power sharing over major decisions, and over workshop structure.^35^ Second, to grapple with the tensions specific to sports and development around research methods that do not resonate with youth sports participants, activities that take away from playing time, and failures to connect youth with the value of YPAR. A focus on new, sports-aligned creative methodologies may help engage participants and create spaces for them to voice their concerns and experiences.^30,36^

This article presents a case study of the use of sports-linked, play-based methods in the initial phase of a YPAR project in the Terai region of Nepal. The goals of this phase were to engage adolescent girls in identifying and planning to address gender-related health challenges using novel play-based methods centred around a sport (cricket), while building their capacity as a youth research team. This article reviews the use of these novel play-based methods to enhance key YPAR processes^35^ and considers their potential to generate relevant data for informing the development of gender-based health interventions.

## Methods

### Setting

The *Cricket Changemakers* study is part of the *Cricket for Equality* programme, a community-based initiative that operates in Nepal’s Morang and Saptari districts, primarily implementing school-based cricket coaching and some showpiece match-play opportunities for women. This programme, developed through a collaboration between Nepali and British sport and development NGOs, started in 2017 in Morang (Biratnagar) and expanded to Saptari in 2021. The *Cricket Changemakers* study applies a YPAR approach to enhance the *Cricket for Equality* project, to promote gender-related transformation and strengthen programme relevance to local adolescents and youth populations.

**Figure 1:**
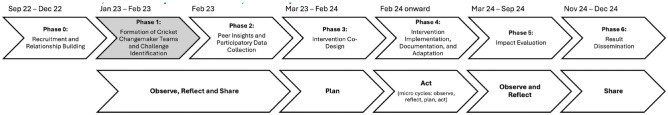
A flowchart illustrating the phases of the Cricket Changemakers project in the Terai region of Nepal. Phase 1 focuses on forming adolescent research teams and identifying gender-related challenges in their communities, while later involve project modifications, action implementation, and impact assessment

This article focuses on Phase 1, the initiation phase, where the objective was to form adolescent research teams, known as the *Cricket Changemakers*, and work with them to identify key gender-related challenges in their communities (Figure 1). This phase aimed to equip these adolescents with the skills necessary for research and action, and to inform later stages of the project, which involve programme modification recommendations based on their findings, as well as engagement in implementation of these modifications and assessment of their impact.

In Morang, the research is centred in the urban municipality of Biratnagar. Biratnagar is the second largest city in Nepal, a commercial hub located in the eastern part of the country, close to the Indian border. While key indicators on gender, health, and educations are not disaggregated to the district or municipality level, Morang is in Koshi province, where data which can be compared to other parts of Nepal is available. Here, school completion rates are typical for Nepal (74% complete Lower Secondary, compared with 73% nationally).^37^ Rates of violence experienced by women here are moderately high; for example, 20.5% of women have experienced physical violence since age 15, however this is still the third highest rate, out of seven provinces.^5^ 30.9% of girls are married before the age of 18.^38^ Notably, these figures are likely to vary widely across Koshi Province. As a province encompassing the three ecological zones of Nepal (mountain, hill, and terai), it is likely that rates are higher in Biratnagar, which is in the terai and where rates of violence and early marriage are universally higher than the other ecological zones.^5^

In Saptari, the research context is more diffuse, covering the municipalities of Rajbiraj (urban), Kalyanpur (rural), and Surunga (rural). Saptari district is in Madhesh Province, which is entirely in the terai and where indicators universally point to significant issues related to gender norms and the health of women and girls. 36.5% of women have experienced physical violence since age 15, compared to a national average of 22.5%. 10.6% have experienced sexual violence (national average, 7.5%), and across all controlling behaviours by intimate partners, the highest rates are reported in Madhesh Province.^5^ Early marriage rates are notably high in the Province; 52.5% of girls are married by age 18.^38^ Other forms of gender-based violence include harassment of girls en route to school and when moving around in public generally.^38^ While school completion rates for boys and girls are largely similar, only 60% of all children complete Lower Secondary (up to class 10).^37^

### Participants and recruitment

To form the Cricket Changemakers teams, adolescent girls from public schools in both districts were recruited. Participants were drawn from eight schools in Morang and Saptari, with a selection process prioritising girls from marginalised backgrounds. A combination of cricketers and non-cricketers were recruited through open invitations at school-based meetings, where students, teachers, parents, and local leaders learned about the project.

Recruitment criteria were girls, in classes 8 to 10 (Lower Secondary), who attended government schools. An all-girl research team was chosen to centre the perspectives of girls in the research process, with plans for engaging boys in later phases of the research. Students were invited from class 8 to class 10 to ensure sufficient interest in the project; however, this resulted in a wide range of ages due to variations in age distribution in schools, particularly in rural areas where girls may start school later or repeat grades.

Interested students were invited to take home parental consent documentation, which was completed and brought in to the first workshop, where participants also completed informed assent. A total of 18 girls attended the initial meeting in Saptari, while 12 girls attended the initial meeting in Morang. In Saptari, the recruiting NGO’s pre-existing relationships with local families allowed for follow-ups with marginalised girls, ensuring their participation. In Morang, recruitment took place through schools, where participation was prioritised based on interest and engagement during the initial meetings.

The initial intention had been for eight girls to form each group of Cricket Changemakers; however, feedback from the collaborating NGOs was that all girls who showed strong interest should be included in the group, and a modification was therefore made to allow this. The Morang group consisted of 9 girls (ages 12–17) from urban public schools in Biratnagar, while the Saptari group included 14 girls (ages 14–21) from rural and urban schools in Rajbiraj, Khadak, and Surunga municipalities. Across both groups, participants from the same schools knew each other but did not know girls from the other schools. Participants came predominantly from indigenous Terai Janajati and Madheshi Dalit communities.

Participants were remunerated for their role in the research; they received travel expenses to attend all workshop activities and would receive a stipend on completion of data collection tasks in later phases of the research.

### Workshop description

Each group took part in workshops in their own district. The workshops were held in spacious indoor meeting rooms, with space for simple in-room games and access to nearby playing fields for more active activities. Facilitation tools such as flipcharts, sticky notes, and coloured pens were prioritised over digital technology, alongside basic cricket equipment including plastic stumps, bats, marker cones, and tennis balls. We (S.B., N.S., A.S., and B.J.) conducted three linked and progressive workshops designed to co-identify a focus for gender-transformative change, develop the participants’ research skills, and enhance their confidence and ability to effect change. Workshops are summarised in Table 1. The facilitators were one female British PhD student, one female Nepalese PhD student, and two members of the Nepalese NGOs, one male and one female. Interactive games and participatory methods were used throughout. Each workshop lasts for 4 h, and all Cricket Changemakers attended all workshops. Workshops took place between 17 January 2023 and 5 February 2023.

**Table 1:** Summary of workshop content, with examples of activities implemented, focusing on play-based activities. Detailed guidance is available at cricketchangemakers.com/resources^39^.

Workshop	Title	Objectives	Example activities
1	Foundations and Context	Develop code of conduct, explore children’s rights, gender sensitisation, introduce the socio-ecological model	• Icebreaker games • Children’s rights game (collecting cones) • Cricket batting activity to represent socio-ecological levels (Box 1)
2	Identifying Challenges and Setting Goals	Identify community challenges for girls, envision future changes, and develop a project goal and research questions	• Game to ‘throw out’ challenges by knocking down stumps • Team-based, collaborative ball passing game for goal setting
3	Preparation and Ethics	Prepare participants for data collection and develop knowledge of research ethics	• Practice of facilitating games for data collection • Discussion on ethical research practices and personal safety

### Data collection and analysis – games to enhance YPAR processes

To reflect on how the workshops, and in particular games, contributed to key YPAR process, we gathered qualitative data captured throughout the workshops: photographs and videos taken during the workshops, and reflective notes of observations and discussions between facilitators immediately following the workshop (S.B., B.J., N.S., and A.S.). Furthermore, at 12 and 18 months after these workshops, we conducted reflective activities with Cricket Changemakers, where as a group they discussed and produced mind maps of the changes they had seen in themselves as a result of their involvement in the research activities.

In order to analyse this data, we (S.B., female British PhD student and N.S., female Nepalese NGO staff member) applied a framework analysis approach, independently coding the textual data (facilitator reflective notes and Cricket Changemaker reflective mind maps) according to their alignment with pre-identified key processes of YPAR (training and practice of research skills, promoting strategic thinking, group work, opportunities for networking, communication, power sharing over major decisions and over workshop structure).^35^ Following Cofie et al.,^40^ we did not rely on quantitative measures to ensure consistency and rigour in our coding but instead used a qualitative-based approach. After independently coding the data, S.B. and N.S. compared codes through dialogue, discussing overlaps and resolving discrepancies, and reviewing visual data to verify key findings. The final narratives were shared with all facilitators for comment and further reflection. This process enhanced reflexivity and ensured that the resulting codes and narratives reflected shared understanding.

### Data collection and analysis – games to generate data

**Figure 2:**
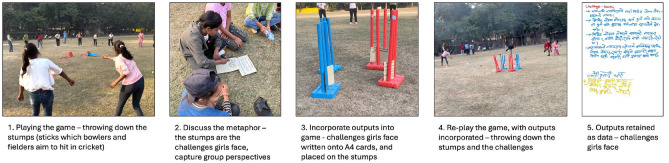
‘Challenge Busters’ game used to identify challenges faced by girls in their communities. Participants throw balls to knock down stumps representing gender-related challenges, followed by discussions and reflections about real-life obstacles

The games used in the workshops apply a play-talk-play approach, with games acting as physical metaphors to shape the “talk” phase of the approach. First, Cricket Changemakers played the game. Then, the metaphor or concept which the game aims to represent is discussed. Next, adolescents discussed their perspectives, views, and observations linked to this metaphor or concept and summarised their discussion by writing their key points on sheets of A4 card. These written materials were then incorporated into the game to consolidate the link between the game and their reflections and observations. An example from one of the games – Challenge Busters – is presented in Figure 2.

Besides games, we used informal creative activities to help Cricket Changemakers express their views and lessons learnt, for example, production of end-of-day summaries using drawings to present to their peers. These drawings and their corresponding explanations were captured through photographs and audio recordings.

The workshops yielded 41 pages of Changemaker-produced summaries, notes, and illustrations outputs, with 28 pages from games and 13 from artistic activities. Where drawings were included, if the explanation for the image was not written next to the drawing, videos and audio recordings were consulted for explanations of the meaning of the drawing. Three distinct sets of data were produced: challenges, goals, and perspectives on empowerment. All data were translated from Nepali into English for analysis, as both members of the analysis team could read English. To ensure consistency in coding, S.B. and N.S. applied a deductive approach, where both independently coded the data into the predefined themes of challenges, goals, and empowerment perspectives. We again compared their coded data through dialogue and consensus, resolving any differences collaboratively.

### Ethical approval

Approval for this study was obtained from the Liverpool School of Tropical Medicine Research Ethics Committee (Ref: 22-031) and the Nepal Health Research Council (Ref: 3641/2022 PhD).

### Facilitator positionality statement

The facilitator co-authors of this study bring diverse perspectives that shape the research design, implementation, and analysis. B.J., a Nepalese PhD student and gender activist and educator from Rautahat, draws on her legal background and lived experience as a woman to provide a gender-sensitive lens to the challenges facing women and girls in Nepal. A.S., the founder of a cricket-for-development NGO, offers local insights from his upbringing in Biratnagar, Morang, and his professional experience in climate change advocacy. He views cricket as a key tool for social change in his community. N.S., a female NGO staff member from Rajbiraj, Saptari, brings firsthand knowledge of community dynamics and the realities of implementing gender-focused programmes in rural Nepal. S.B., a British PhD student with a background in cricket coaching and sport-for-development research, offers an external perspective, drawing on her work in global contexts while remaining mindful of her outsider status.

## Results

**Figure 3:**
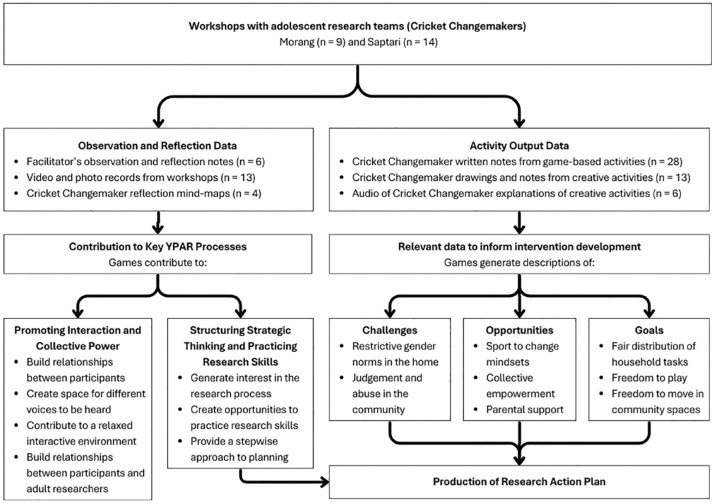
A diagram depicting the different types of data collected during the workshops, including observational data, play-based tasks, and creative outputs. The figure highlights how games were used to generate data that informed the development of research action plans, and how games contributed to YPAR processes to support development of those action plans

As shown in Figure 3, the data collected through the workshops included both observational/reflection data and outputs from activity-based tasks. Games contributed to key YPAR processes by promoting interaction and collective power. Through game-based methods, the Cricket Changemakers identified challenges, opportunities, and goals related to gender equity. This structured use of games, with direct links to the information needed to inform development of a research action plan, supported both strategic thinking and practical development of research skills, and contributed to the development of adolescent-led research action plans.

### Play-based methods promote interaction and collective power

The workshops effectively used structured play-based methods to enhance key YPAR processes related to group work, opportunities for networking, communication, and power sharing. Facilitators and Cricket Changemakers alike reflected on the workshops’ impact, observing increased engagement, empowerment, and collaboration.

#### Building relationships and networks

The workshops fostered a sense of teamwork and collaboration, with facilitators noting that group dynamics were enhanced through the diverse range of activities. The use of games as icebreakers and tools for rapport-building was particularly effective. For instance, a game involving a race to grab a ball introduced an element of friendly competition, encouraging celebration and vocal self-expression. This shift was visible in photos, where serious expressions were replaced by smiles and laughter after these games.

**Figure 4:**
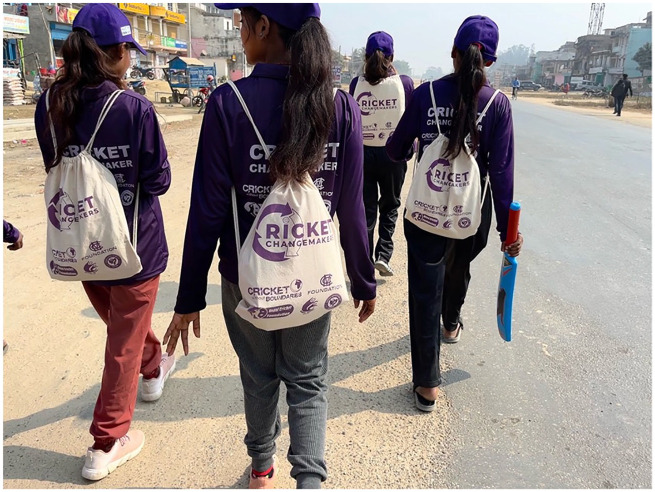
Photograph of Cricket Changemakers from Saptari districts. The image captures the participants engaging in informal networking while walking to the playing fields, reflecting the development of group dynamics and team identity

Facilitators further noted that the journey from the meeting hall to the playing fields offered informal opportunities for networking and relationship-building. Dressed in matching jerseys and carrying cricket equipment, the Changemakers engaged in lively conversation, which fostered a sense of unity and collective identity (Figure 4).

In Morang, the Cricket Changemakers underscored the importance of group work and networking in building relationships between themselves that connected to their external lives; this helped form a strong inter-school network: ‘We connected with new students, we came to build connections between schools via Changemakers’. In contrast, the Changemakers in Saptari focused on internal group collaboration, highlighting how group work fostered mutual support and strengthened their sense of responsibility in achieving the goals of the project ‘We introduced to each other, this led to development of supportive feelings, that is why it is easy to work if there is mutual support. We increased friendship [through these activities], a development of collective feelings’.

#### Fostering communication

Facilitators observed that games were instrumental in promoting open communication and breaking down hierarchical barriers. Participants, who initially appeared reserved, became more relaxed and vocal after game-play, with quieter members contributing more actively in smaller groups. In Saptari, facilitators noted that girls from the most rural areas and most marginalised groups were in particular more engaged and keen to communicate during game-play led discussions. In Morang, the difference was notable in girls who were more interested in the sports element of the activities – appearing disinterested during periods where the groups sat down, these girls were eager to have the first hit, throw, or catch in the games and were actively engaged in the conversations around the games.

In Morang, participants reflected on how the workshops improved their communication skills, particularly in their ability to advocate for gender equality ‘We became outspoken and confident [and] advocated for gender equality’. In Saptari, communication was closely tied to relationship-building, as the interactive nature of the workshops encouraged quieter participants to articulate their ideas clearly. The more confident Changemakers noted the positive changes they saw in their quieter peers, emphasising that this enhanced communication built trust and laid the foundation for stronger collaboration.

#### Power sharing and leadership development

Facilitators reflected on how the games created a conducive environment for power sharing. The workshops, held in settings typically viewed as formal, were transformed into interactive spaces where participants felt comfortable expressing themselves and taking ownership of decision-making and emphasised the collaborative nature of the work they were to undertake. Games such as knocking down objects symbolising challenges not only engaged the participants but also fostered discussions on overcoming real-life obstacles collectively. Finally, facilitators note that the blend of activities saw different Cricket Changemakers voices being centred at different times; in particular, girls in both contexts who were less engaged or willing to speak when sat at a table became lively, more engaged, and contributed to peer-to-peer discussions, supporting power sharing between the Cricket Changemakers themselves.

In Morang, the Changemakers felt empowered by their involvement in the activities, independently driving change in their school from their involvement in the research and indicating ownership over the project; ‘We got inspired and empowered ourselves, whatever I learnt from the research we have shared with others in school, we became Changemakers’ while in Saptari, the emphasis was on the creation of the team that would work together for the remainder of the research project: ‘Establishment of a friendship bond, it creates bond between players, coach, (researcher name) and also Changemakers, and relationship growth’.

### Challenges, goals, and opportunities for transformation

The Cricket Changemakers, through the play-based activities, highlighted key challenges that centred on gender inequality, particularly societal norms that restrict girls’ participation in sports like cricket and limit their educational opportunities. The games that focused on defining challenges consistently surfaced themes of exclusion and rigid gender expectations. The Changemakers expressed frustration with societal norms that confine girls to domestic roles, restrict their personal growth, and devalue their education:

*Parents do discrimination as boys are left to roam around without any work and girls must do household work, stay at home and have no permit to read at home. And if we ask permission to go school to read and write as others, they comment as, ‘Why do you need to study? After all you will be doing household work after marriage’.* (P. Morang)

**Figure 5:**
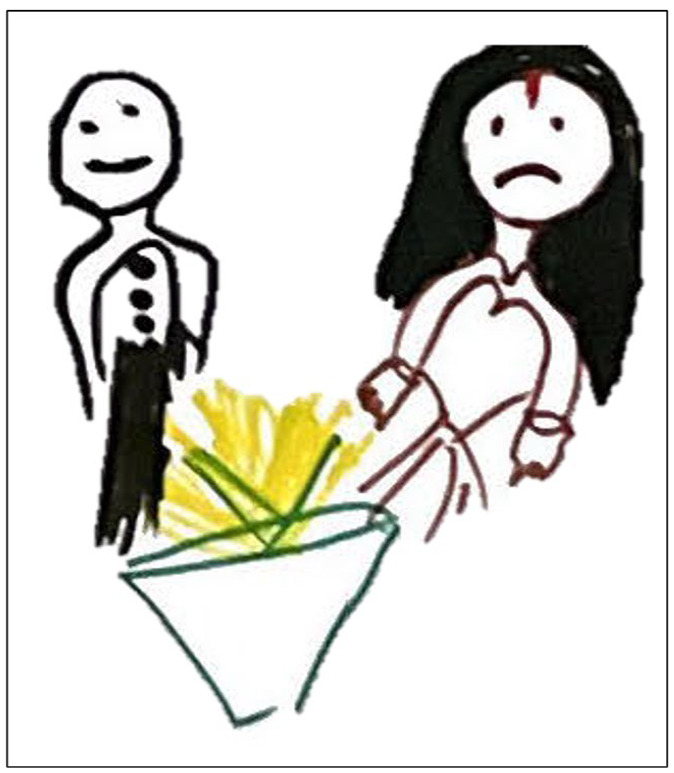
A visual representation of the barriers to mobility faced by girls in Saptari district. Drawings created by the Changemakers highlight double standards and the limitations on girls’ freedom of movement compared to boys

The uneven distribution of household chores was a key issue in Morang, while girls in Saptari particularly expressed their frustration over the double standards that affect their freedom compared to boys (Figure 5). This group emphasised the limitations put on girls’ mobility, and negative experiences moving through community spaces:

*Compared to boys, we reach home early. We must take permission from parents to go out the home. We have more responsibility towards parents and the home than boys.* (S. Saptari)

Despite these barriers, many Changemakers were already challenging traditional gender roles from within their communities. However, resistance to change was met with negative attitudes, including instances of physical aggression from boys who sought to reinforce traditional norms:

*While [we] go to play any tournament or any game, society bullies us. The bad societal thoughts and practice will always try to pull back the Changemakers in society.* (G. Saptari)

In goal-setting activities, the Changemakers expressed a clear desire for gender equality in both sports and household duties. They challenged the notion that girls should be confined to domestic work, advocating instead for shared responsibilities between genders:

*Boys must also do household work, and girls can also get involved in professional work. It’s easier and faster if boys also do the household chores.* (A. Morang)

The Changemakers envisioned a future where community perceptions of girls’ capabilities are transformed through their achievements, and girls believed that showcasing success stories and role models could help change these perceptions and garner family and community support:

*While playing cricket, many people of different communities observed our game and effort. They now do not tease and comment on other girls of their community. Change in their mindset has been seen*. (R. Saptari)

**Figure 6:**
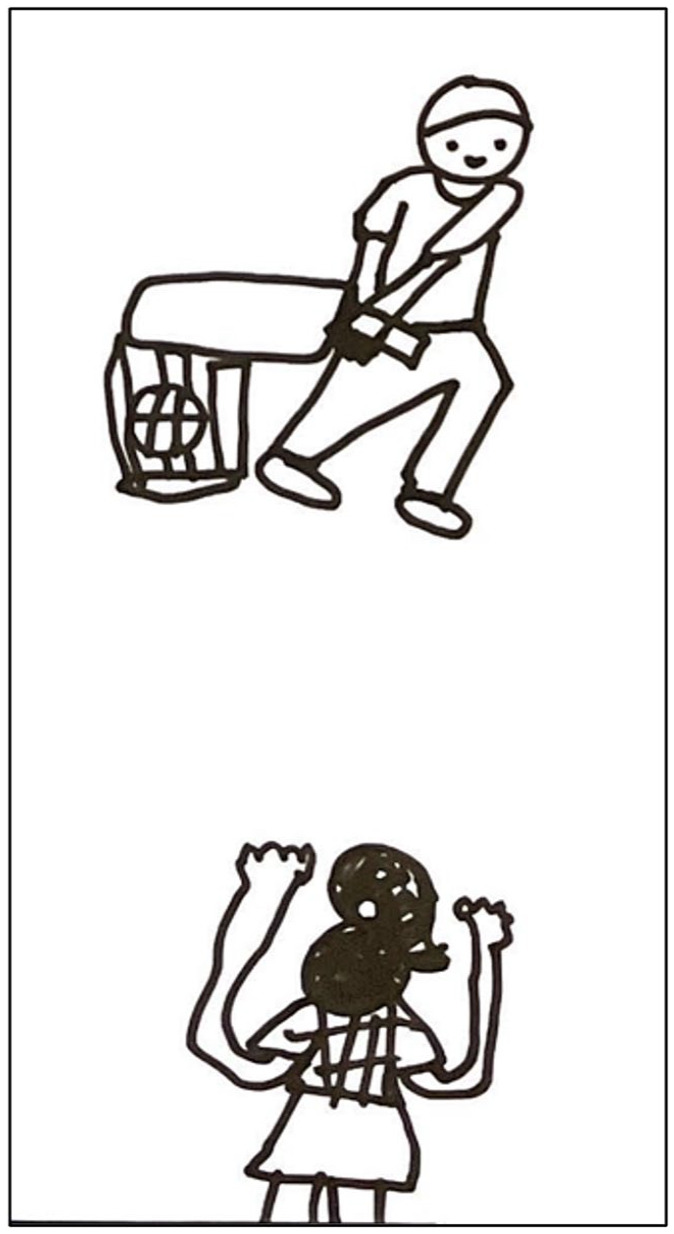
Artwork produced by participants that illustrates the role of cricket in building confidence and challenging societal expectations for girls. The image showcases how participants envision cricket as a tool for empowerment and gender transformation in their communities

Cricket was seen not only as a sport but as a tool for empowerment, self-efficacy, and broadening life opportunities (Figure 6). Their ultimate goal was to use cricket to build confidence and agency among girls, challenging societal expectations that question girls’ engagement in sports:

*We will generate self-power by playing cricket which will enable parents not to comment that, ‘Your study is disturbed or being poor and you cannot achieve any by playing cricket’.* (N. Saptari)

The Cricket Changemakers viewed this empowerment as rooted in both realistic assessments of their current conditions and a visionary outlook for the future. Resilience, self-belief, and peer support were central to their sense of empowerment:

*My own self confidence encourages me and makes me strong. Observing, listening while outside encourages me to think that I can also do . . . Listening to new lessons helps me to be strong.* (S. Saptari)

They attributed their growing confidence to the encouragement of supportive families, role models, and peers, as well as their involvement in sports, which helped develop both mental and physical strength.

### Strategic thinking: Developing research action plans

At the end of the second workshop, the Cricket Changemakers outlined their project objectives and research questions for the upcoming year, focusing on understanding the challenges and opportunities tied to their goals. Despite working as two distinct groups, the Morang and Saptari teams set closely aligned goals:

- Saptari’s objective: To build girls’ confidence and alter community perceptions about girls’ roles using cricket.
- Morang’s objective: To increase girls’ participation in cricket.

To further explore these issues, the Cricket Changemakers will undertake peer-to-peer data collection in Phase 2 of the study. Collaborating with facilitators, they mapped out objectives and developed a research plan. Together, the groups identified two key research questions, discussing which questions would best deepen their understanding of the context and guide their future actions. In Saptari, they planned to ask: ‘Why don’t girls play cricket?’ and ‘What happens when girls play cricket?’. Meanwhile, in Biratnagar, the questions were: ‘What stops girls from playing cricket?’ and ‘What helps girls to play cricket?’. Although both sets of questions aim to uncover similar insights, Saptari’s questions emphasise understanding the lived experiences of adolescents, and in particular girls.

#### Structuring strategic thinking and practicing research skills

Facilitators observed that the structured, stepwise application of games allowed the Cricket Changemakers to systematically identify key issues and develop co-created research questions. There was a strong link between the metaphors used in the games and the role that data would play in informing the creation of research questions; this was particularly true of the game ‘Changemakers Cricket’ (Box 1), where the theoretical framework of the Socio-Ecological Model was introduced – the layers of the models would be continuously referenced by the Cricket Changemakers throughout the workshops. The workshops also provided practical opportunities for participants to engage in research activities, such as data collection, which developed their interest in research. In both Morang and Saptari, the Changemakers highlighted that the games helped them practice collecting and reflecting on data, while also developing key research skills they would apply in subsequent phases of the project.

**Box 1.** Changemaker Cricket – understanding our strengths and power for change.

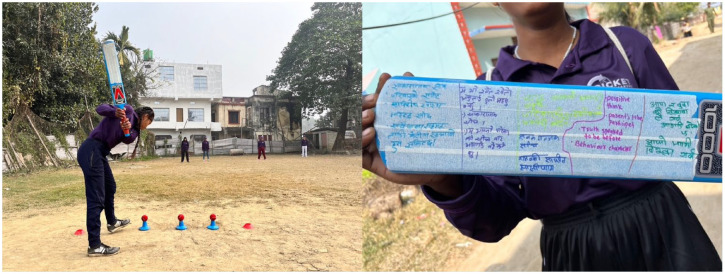 In this batting game, we used different coloured lines, marked by cones, progressively further from the batter (the individual) through which the balls had to be hit, with each line representing the interpersonal, community, and societal levels. We discussed the power of working together for change (using two hands to hold the bat while batting) and using our full power to hit the balls, as well as the barriers (represented by fielders aiming to stop the balls) we face at each level. Between rounds of the game, we wrote our collective strengths/powers on the bat to consolidate the Cricket Changemakers’ understanding of their existing potential to make change and discussed ideas of how to remove fielders (i.e. overcome challenges at each level).

Reflecting on their experiences, the Morang Cricket Changemakers highlighted the role of interactivity in driving interest in both research and in playing cricket; they linked together ‘More interactive way or good use of the creativity’ to ‘Interest in research and deeper engagement’ and ‘Increased interest in cricket’. The Saptari group noted that the workshops fostered their ability to think strategically. For these participants, strategic thinking was linked to the gradual development of personal and group capacities, with the support of teachers and coaches, to collaboratively reflect on how to plan a collective way forward. ‘We had established our bond of friendship, by describing [our] reality we got support [from others], we incrementally developed and achieved our expected goals, we had real understanding between facilitators, Changermakers and coaches’.

### Methodological limitations

One key limitation of these methods was the balance between play-based activities and discussion. While the games fostered engagement and interaction, they may have limited the depth of conversations compared to traditional focus group discussions, as participants spent a significant portion of time playing rather than engaging in more complex reflections. This was particularly true in Morang, where the younger age profile may have contributed to more superficial levels of engagement with the questions posed as part of the games, with often briefer responses recorded, particularly in the game related to empowerment. Exploring these through conversations after the documentation allowed for more detailed engagement, but these were not audio recorded. A key recommendation for future use of these methods is to include audio recording of these follow-up conversations.

In addition, which voices were documented often depended on the individual responsible for writing, potentially influencing the recorded data. This was particularly true in Saptari, where three Cricket Changemakers from the school nearest to the collaborating NGO office, who had some experience of youth leadership activities, often were the first to pick up the pen or describe and explain their group responses. Early identification of these intra-group dynamics meant that by workshop 2 and 3 the facilitating team made a particular effort to distribute roles to others in the group. Despite these constraints, the workshops were effective in fostering leadership, teamwork, and collective responsibility among participants, as highlighted by the emphasis in Cricket Changemakers reflections on the role of these early workshops on the creation of a cohesive team and on the growth of their peers’ confidence and communication skills.

Finally, when coming to analysis of the output data, S.B. and N.S. observed that it was not always possible to disaggregate or analyse data based on key characteristics such as age, religion, or caste/ethnicity, as groups contributed to many of the outputs collectively. This meant that subtleties in differences in experiences between these groups could not be examined.

## Discussion

This study proposes an integrated approach to delivering YPAR workshops, blending sports, discussion, and creative methodologies to create an engaging environment to catalyse new thinking in public health intervention design. The strategic use of games went beyond traditional ice-breaking roles, serving as both a data collection method and a tool to demystify the research process. This approach aligns with growing recognition of the need to engage young people and adolescents in public health planning for issues that affect them.^41–43^ Our findings suggest that integrating sports into youth participatory research can add value to the repertoire of methods used in the field of YPAR. These methods can tap into the interests of diverse adolescents, and may engage and centre the voices of different young people to those who involved in more conventional method research projects. The enthusiasm and engagement observed throughout the workshops underscores the potential of play-based methods to both involve and empower adolescents as co-researchers in public health.

The integration of game-based methods within this YPAR project provided a robust framework for adolescent girls in Nepal to articulate the challenges and aspirations shaping their lives. These methods fostered a reflective discourse, empowering participants to move from recognition to planning actionable next steps in their roles as co-researchers. The importance of participatory methods in promoting meaningful community and co-researcher engagement is well documented^44,45^ and was evident in the energy sustained throughout our sessions. Notably, the use of physical games was key in the relatively rapid formation of trust and rapport, both between co-researchers and, importantly, between adult and adolescents. This is a crucial element of effective YPAR^46^ and we have since observed that this trust continued to develop throughout the research process. This accelerated bonding also supported collective recognition of shared struggles, such as the disproportionate burden of domestic work faced by girls. This is a challenge which has been identified and addressed in interventions within the region, such as early adolescent school-based interventions on gender norms in Nepal’s Terai region,^11^ and interventions promoting positive masculinity in male cricketers in neighbouring Bihar state, India.^12^

While health outcomes were not the primary focus defined by the Cricket Changemakers, the surfacing of themes relating to gender-based violence and empowerment in decision-making underscores the inextricable link between gender norms and health. The articulated goals and empowerment narratives related to challenges emphasise the Changemakers’ pre-existing knowledge of the social norms that constrain their participation, their determination for change, acknowledgement of their own assets for change, and recognition of the need for support from social agents to affect change. Our research suggests that girls are acutely aware of the need for ‘power with’ their peers, schools, and family support networks, and view this as part of their own empowerment, aligning with emerging fields of thought that emphasise ‘power with’ as a relational dimension of empowerment and social power.^47,48^

The decision to use cricket as a focus in this research, rather than generic bat-and-ball games, was intentional and rooted in practical considerations. While the games played during the workshops were adapted from cricket training drills and did not involve full matches, cricket-based activities were chosen because of their potential for long-term engagement and sustainability. First, sports like cricket offer opportunities for participants to develop specific skills and competencies that foster personal growth and empowerment, as seen in the results where the Changemakers expressed pride in their ability to demonstrate physical competence and challenge gender norms. This connection to a recognised sport enhances the value participants, their parents, and their community place on their achievements. Second, linking the activities to cricket introduces potential partnerships with local cricket associations, who are invested in sustaining sports programmes beyond the life cycle of NGO funding. This alignment with local sporting bodies allows for the mainstreaming of successful interventions, embedding gender-transformative practices or other health promotion initiatives into community-based sports activities. Examples of this being actioned in cricket include heart health screening in Uganda^49^ and refugee inclusion in countries across Africa and Europe.^50,51^

### Limitations and future directions

While the study’s insights are drawn from a cohort with substantial representation from marginalised groups, particularly the Madheshi Dalit community, the high representation from the Terai Janajati, who’s communities provide a great deal of more freedom to girls to participate in sports, means that it will be vital for the Cricket Changemakers research teams to engage with a wider group when gathering further information about identified challenges before recommending adaptations to the Cricket for Equality programme, to ensure the intervention’s wider applicability.

The workshops’ success was partly due to the dual expertise of the lead facilitator in both cricket coaching and research, a model that may pose scalability challenges. Future work should explore the development of facilitation capacities among community sports coaches or researchers, or the viability of co-facilitator models, based on workshop facilitation materials made available online.^39^

The workshops’ overall goal, to engage adolescent girls in identifying and planning to address gender-related health challenges using creative, play-based methods, was ultimately supported by the data and outputs from these workshops. There are, however, areas that could have been improved or modified to strengthen this phase of the research. The project’s cricket-centric orientation may have limited the focus of the identified challenges, goals, and research questions. Similarly, by focusing on the challenges girls face in a broad sense, we ended up focused on gender transformation. A non-gendered call for challenges (e.g. what challenges do young people in your community face) or a more specific focus on health could support adolescents to identify other ways that sports-based interventions can support health promotion. Finally, the focus on interpersonal and community levels inadvertently led to a shortfall in insights into institutional and policy-level influences, a gap that future work could aim to address.

Nevertheless, the commentary around empowerment from the Cricket Changemakers establishes a valid link to the transformative potential of sports, as posited by the ‘Sport for Development’ concept, and over time, the Cricket Changemakers’ focus might organically evolve to encompass more nuanced or varied public health challenges. As this YPAR cycle progresses, it has the potential to inform adolescent-led health interventions that can be mainstreamed into policies and practices beyond the health system. This potential for sports organisations to serve as vehicles for health promotion, especially among audiences less engaged with formal health systems, presents an exciting opportunity for integrated public health strategies.

## Conclusion

The Cricket Changemakers project contributes a novel approach to the fields of creative health promotion and gender-transformative research. It underscores the potential of participatory, play-based methods in mobilising adolescents for social change and offers actionable insights for practitioners, researchers, and policymakers. The integration of sports and participatory research not only enriches the landscape of methods in YPAR but also leverages existing social structures to advocate for health and gender equity.
